# The use of artificial intelligence in healthcare as perceived by the citizens and patients: a narrative review of the literature

**DOI:** 10.1093/eurpub/ckaf189

**Published:** 2025-11-14

**Authors:** Fabiana Nuccetelli, Valeria Gabellone, Francesca Marsano, Francesca Giovanetti, Pietro Dri, Maria Rosa Valetto, Rosa Prato

**Affiliations:** Department of Experimental Medicine, University of Salento, Lecce, Italy; Department of Experimental Medicine, University of Salento, Lecce, Italy; Zadig Ltd Benefit Company, Milan, Italy; Italian Society of Artificial Intelligence in Medicine (SIIAM), Rome, Italy; Zadig Ltd Benefit Company, Milan, Italy; Zadig Ltd Benefit Company, Milan, Italy; Department of Medical and Surgical Sciences, University of Foggia, Foggia, Italy; Hygiene Unit, Policlinico Foggia Hospital, Foggia, Italy

## Abstract

The growth of scientific literature on large language models (LLMs), such as ChatGPT, anticipates their central role for accessing health information but poses potential risks, including the false belief that artificial intelligence (AI) could replace doctors in providing reliable information. Our study, part of the Slow AI project launched in partnership with the Slow Medicine ETS Association, reviewed the literature on ChatGPT use by the public, analyzing citizens’ and patients’ perceptions of using AI for health-related questions, identifying key benefits and concerns, and providing recommendations for the safe and effective use of LLMs. We conducted a narrative review following PRISMA guidelines, including qualitative, quantitative, and mixed-methods studies, selected through a search of the PubMed database. Data were extracted and analyzed using a predefined form. Out of 388 records, 120 studies were included, primarily from the USA (65), Europe (19), and Asia (15). Most studies focused on general medicine (37), with patients (57) being the main participants. Key findings include that LLMs improve access to health information, aiding diagnostic accuracy and patient understanding. However, risks exist, such as inaccurate or outdated information, lack of empathy, and privacy concerns. These challenges highlight the need for reliable AI training with real-world data and clinician oversight to mitigate risks. Lastly, while LLMs can improve communication, they should complement, not replace human interaction. LLMs in healthcare offer great potential but also present risks. Safeguards and clinician oversight are crucial to preserve patient safety and doctor-patient relationship.

## Introduction

The scientific literature is growing rapidly with studies on the use of large language models (LLMs), with ChatGPT being the most widely known, as a dominant future tool for accessing health information. It is anticipated that more people will turn to ChatGPT and similar tools to ask questions about their health, such as listing symptoms for diagnostic guidance, seeking interpretation of test results, looking for treatment advice, or exploring new therapies for their conditions [1, 2].

This approach, while democratizing access to health information, carries significant risks for the lay public, including the misconception that artificial intelligence (AI) could replace a healthcare provider or even give more reliable and up-to-date health information [3]. This trend is already present, as evidenced by tools like Babylon in the UK healthcare system, where citizens can choose between consulting an AI-driven diagnostic pathway or a general practitioner as their first-line contact for health concerns, with the former offering significant cost-saving opportunities [4].

Analyzing the impact of AI on public health, particularly the risks and potential benefits of AI use alongside medical professionals, can guide a proper use of ChatGPT and other LLMs and integrate these tools in the framework of measured, respectful, and equitable medicine.

Based on these premises, a narrative review of the scientific literature on the use of ChatGPT by patients and general population was conducted as part of the Slow AI project, designed and launched in partnership with Choosing Wisely Italy/Slow Medicine ETS. The objective was to collect information on the use of LLMs for health questions, highlighting both the critical concerns and benefits of using ChatGPT, and defining some recommendations for use by the general population.

Specifically, the review explored current advancements in areas such as the role of ChatGPT in expanding public access to health information, the risks of misinformation, measures to ensure the accuracy and reliability of AI-provided health information, impacts on the patient-provider relationship, and the broader societal implications of AI integration in healthcare.

## Methods

### Objective of the review and key areas of interest

We were primarily interested in the perceptions and experiences of citizens and patients on use of LLMs in health information. In particular, the narrative review aimed to investigate the current state-of-the-art regarding predefined relevant thematic categories such as the role of ChatGPT in enhancing public access to health information, the potential risks of misinformation, the measures to be implemented to ensure the accuracy and reliability of health information provided by LLMs, the impact on patient-provider relationships, and the broader societal implications of widespread AI utilization in healthcare.

For our study, a specific PECO question was formulated: “Which is the impact of health information provided by AI-driven LLMs on patient information, patient-provider relationships and the broader social landscape, as perceived by citizens and patients?”

### Criteria for considering studies for this review

#### Types of studies

This is a narrative review conducted following the Preferred Reporting Items for Systematic Reviews and Meta-Analyses (PRISMA) guidelines. We included all studies that used qualitative and quantitative methods for both data collection and data analysis. We also included mixed methods studies where it was possible to extract data derived from qualitative research. We included studies both interventional and non-interventional studies.

#### Inclusion and exclusion criteria

The inclusion and exclusion criteria adopted to select studies are reported in Table 1.

**Table 1.** Inclusion/exclusion criteria adopted to select studies

Study	Inclusion criteria	Exclusion criteria
Population	All potential LLMs users for health information: citizens (and subgroup of citizens), patients, and/or healthcare professionals and their experiences and perceptions.	Technical or algorithmic aspects not exploring human experiences and perceptions of LLMs in a healthcare context.
Setting	Healthcare environments (e.g., hospitals, clinics, public health centers), both public and private, where LLMs are used to access health information.	Non-healthcare contexts (e.g., education, entertainment) or general use of LLMs without a clear healthcare focus.
Study design	Quantitative, qualitative, or mixed-methods studies, including observational studies, experimental research, systematic reviews, and meta-analyses. No restriction on sample size.	Single case studies or studies with unclear or unreported methodology. Preprints or publications lacking methodological transparency. Non-peer-reviewed articles (e.g., opinion pieces, unverifiable reports).
Outcomes of interest	LLMs role in improving access to health information. Quality, reliability, and usefulness of the information provided. Risks of misinformation and measures to ensure information accuracy. Impact on patient-provider relationships. Social and ethical implications of LLMs use in healthcare.	AI applications in healthcare without specific reference to LLMs.
Language	Studies published in English or Italian.	Studies published in other languages or that cannot be effectively translated.
Publication date	Studies published up to May 31, 2024 (no retrospective restrictions indicated).	Studies published before the relevant timeframe (if later specified).
Geographical area	No geographical restrictions.	Unidentified or unreported geographical context.

### Search methods for identification of studies and data management

#### Electronic searches

We searched the PubMed electronic database for eligible studies by developing a specific search strategy. We chose this database because we anticipated that it would provide the highest yield of results based on preliminary and exploratory searches. There were no time, language, or geographic area restrictions for the search. The following search string was used: “(LLM) AND (health) AND [(patient) OR (people) OR (general population) OR (citizen) OR (person)].”

#### Selection of studies

**Figure 1.**
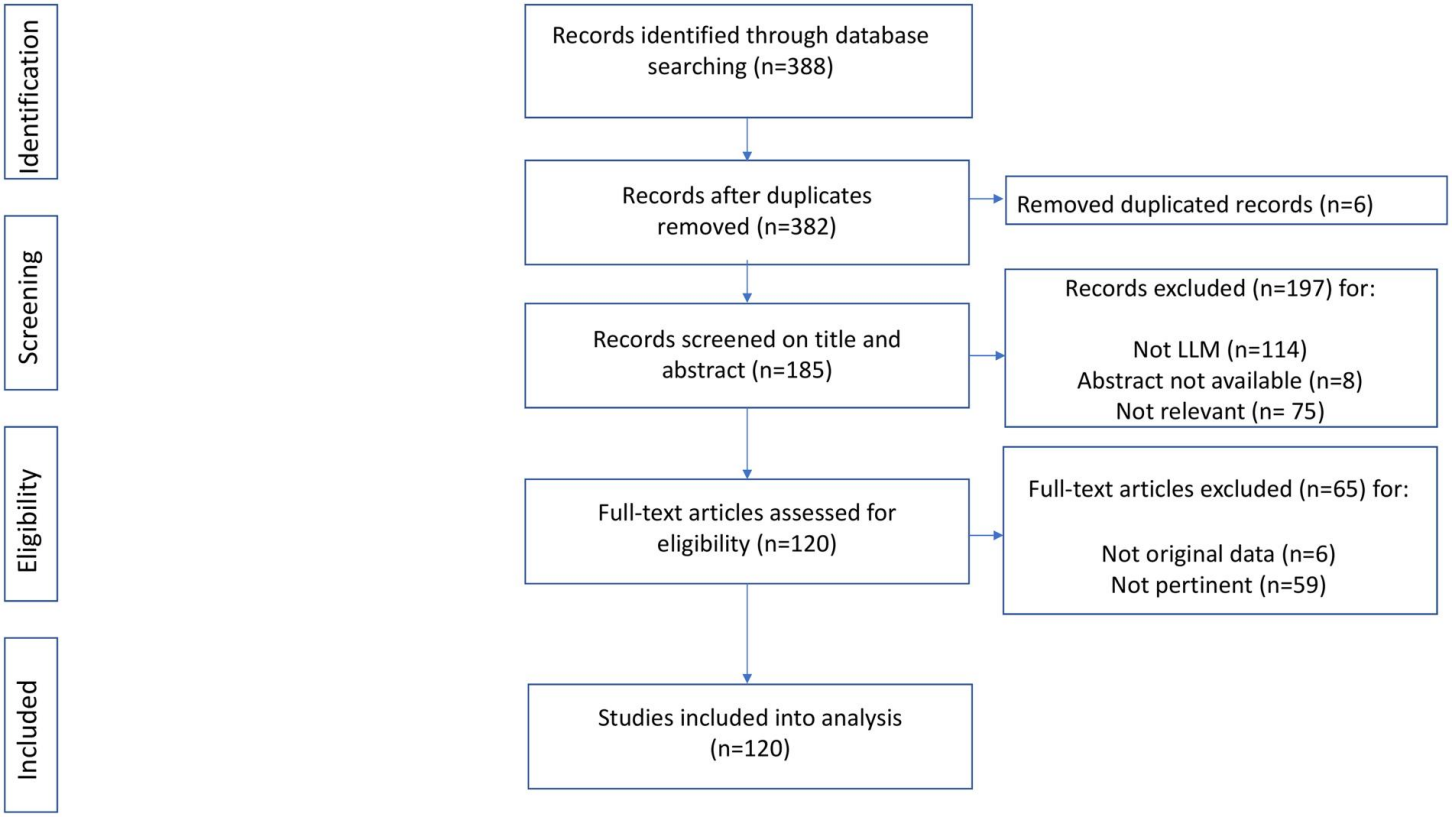
Study flow diagram.

We collated records identified on the PubMed list into a citation manager database and removed duplicates. Two experts independently assessed each title and abstract of the identified records to evaluate eligibility. At this stage, we discarded abstracts that were clearly irrelevant to the topic of this review. We retrieved the full texts of all abstracts identified as potentially relevant. Two review authors independently assessed each full-text article for inclusion, according to predefined criteria and thematic categories. For both the title/abstract and full-text screening, we resolved disagreements by discussion or involving, when required, a third review author. We included a PRISMA flow diagram to show our search results and the process of screening and selecting studies for inclusion (Fig. 1).

For titles and abstracts published in languages not spoken by any member of the review team (i.e. languages other than English), such as Chinese, Japanese, Russian, Arabic, Hindi, and Turkish, we used open-source translation software (Google Translate) to conduct an initial screening and assess eligibility. If a potentially relevant article was not available in full-text in a language we could read or reliably translate, it was excluded from the review due to the impossibility of adequately assess its content and methodological quality.

### Data extraction

We used a data extraction form designed for this review. From each study, we extracted information on title, authors, journal, publication date, geographical area, type of study, language and healthcare setting. Additionally, we collected all data relevant to the objective of the review, including descriptions of thematics categories such as LLMs role in improving access to health information and main outcomes, potential risks and limitations of LLMs, measures to be implemented to ensure reliability of health information provided by LLMs, impact on patient/provider relationships and social implications. One author performed data extraction for all included studies, and two additional authors independently checked the extracted data to ensure completeness and accuracy.

### Data analysis and synthesis

Given the high number of descriptive data we planned to collect, we decided to analyse and synthesize the qualitative evidence using a thematic synthesis method, one of several approaches recommended by the Cochrane Qualitative and Implementation Methods Group [5], that is particularly appropriate for this type of data as it offers a clear and systematic framework for assessing studies with qualitative, and mixed-methods.

For data extraction and synthesis, we followed this process: first, two authors chose the articles deemed most consistent to the objectives of the review. Then, two other authors selected the articles deemed most consistent with the review objectives. Then, two other authors verified the extracted data and supplemented or completed as needed. During this process, we coded only those data that we judged as directly relevant to the topic of the review; when in doubt about the relevance of the data, we chose to code them. We then synthesized these data into the four predefined thematic categories [(i) LLMs role in improving access to health information and main outcomes; (ii) Potential risks and limitations of LLMs; (iii) Measures to be implemented to ensure reliability of health information provided by LLMs; (iv) Impact on patient/provider relationships and social implications] to carry out the results of the review. Once the results were drafted, we shared them with all the authors. Finally, we reread the included studies to verify that we had extracted all data relevant to the findings.

## Results

### Results of the search

In total, 388 records were identified on the PubMed database. After the screening of titles and abstracts and the assessment of the full-text articles, 120 studies were included in the analysis (Fig. 1).

The full list of these studies is reported in the Supplementary Material (Table S1).

### Characteristics of the studies

For a detailed description of the studies that we included and sampled, see Table 2.

**Table 2.** Characteristics of the selected studies

Type of study	*N*
Qualitative survey	47
Quantitative survey	26
Review	19
Observational study	11
Other	17
Population	
Patients	57
Healthcare workers and patients	20
Healthcare workers	18
Students	13
General population	4
Other	8
Geographic area	
America	65
Europa	19
Asia	15
Intercontinental	12
UK	4
Canada	4
Oceania	1
Medical area	
General medicine	37
Orthopedics	11
Neurosurgery	10
Ophthalmology	7
Gastroenterology	7
Cardiology	5
Oncology	4
Categories	
LLMs role in improving access to health information and main outcomes	106
Potential risks and limitations of LLMs	88
Measures to be implemented to ensure reliability of health information provided by LLMs	78
Impact on patient/provider relationships and social implications	75

### Types of studies

The largest part of the studies used a qualitative methodological approach (47). The remaining part of the studies included quantitative studies (26), reviews (19), papers (11), and other studies (17).

### Studies’ settings

#### Population

In all studies, the authors sought the perspectives and views of the population subgroups by distinguishing and categorizing them into groups during the analysis. Most of these studies included patients (57), followed by healthcare workers and patients together (20), only healthcare workers (18), general population (13), students (4), and others (8).

#### Geographic areas

The majority of studies came from the USA (65) and Europe (19), the others were from Asia (15), UK (4), Canada (4) and Oceania (1); 12 studies collected intercontinental data.

#### Medical areas

The 120 selected studies include a diversified medical background, identifying a growing interest in LLMs in all areas of medicine. Most of the articles analysed were from the fields of general medicine (37), orthopedics (11) and neurosurgery (10), followed by ophthalmology (7), gastroenterology (7), cardiology (5) and oncology (4).

### Key findings on the impact, limitations, and safety of LLMs in healthcare

#### LLMs role in improving access to health information and main outcomes

Most of the selected studies examined the role of LLMs in health information. With regard to the accuracy of health information provided by LLMs, several studies demonstrated the importance of LLMs in improving the speed and accuracy of diagnosis and disease screening. The high performance of LLMs in terms of relevance, clarity and emotional sensitivity was also highlighted. In addition, the use of LLMs was thought to enable patients to take more control of their health care and to better understand the evolution of their health needs.

#### Potential risks and limitations of LLMs

Of the 120 selected studies, 88 analysed the potential risks and limitations of LLMs in healthcare. Overconfidence in LLMs-based systems was recognized as a risk also to the medical profession with the likelihood of biased decision-making and inaccurate communication. Several analysed studies also brought out several concerns, including issues related to data distortion [6–8] with cybersecurity breaches and recommended further research. In more detail, they raised the point that the use of unregulated cloud services that compromise data security, exposure of sensitive patient data, confidentiality breaches, fraudulent use of information, vulnerabilities in data storage and communication, and inappropriate access or use of data, accentuate the risk of patient misinformation [2, 9, 10]. In addition, a study delved into the challenges and ethical considerations of integrating LLMs, potential biases, and regulatory compliance, emphasizing the need for a balanced, prudent, and thoughtful approach prioritizing citizens’ safety and ethical standards [11].

#### Measures to be implemented to ensure reliability of health information provided by LLMs

Many of the reviewed studies focused on measures to be implemented and suggestions to ensure the reliability of the health information provided by LLMs, in order to overcome what has been identified as one of the most relevant potential risks of using AI in healthcare. Following a careful consideration of both the opportunities and risks of today’s AI chatbots, the authors recalled the necessity to develop appropriate guidelines and best practices for their use as a source of health information to ensure the safe and responsible use of LLMs in healthcare settings [12, 13].

Some reviewed studies highlighted the need for rigorous clinical validation and testing to ensure that LLMs meet the standards of evidence-based medicine and suggested that, to this aim, regulatory bodies should develop robust standards to ensure the safety, efficacy, and ethical use of LLMs. Collected data indicated that this process includes defining what constitutes acceptable accuracy for different AI applications, ensuring transparency in algorithms, and protecting patient privacy [1, 14, 15].

#### Impact on patient/provider relationships and social implications

A study called for efforts to develop artificial empathy noting that while such efforts are laudable, they should aim to complement rather than replace human empathy, in order to avoid further isolating patients and undermining the fundamental therapeutic alliance between patients and physicians [16]. Another study considered the impact of LLMs on fundamental aspects of the patient-provider relationship and the underlying importance of a synergistic relationship between LLMs and providers [17]. Other studies highlighted the need to adopt a critical awareness approach to the implementation of LLMs in healthcare, applying critical thinking and reasoning. In line with this reasoning, they emphasized the importance of preserving the core values of the doctor-patient relationship, such as trust and honesty, conveyed through open and honest communication [18–20].

Examples of relevant conclusions drawn from the studies selected according to the four thematic categories are reported in Table 3.

**Table 3.** Relevant conclusions drawn from the studies selected according to the four predefined thematic categories

Finding	Citation	Reference
LLMs role in improving access to health information and main outcomes	“The story taken using LLM showed an overall accuracy of 44% in identifying the most likely diagnosis and 55% in suggesting the indicated medical provisions.”	[21] Abi-Rafeh J, Mroueh VJ, Bassiri-Tehrani B, *et al.* Complications following body contouring: Performance validation of bard, a novel AI Large Language Model, in triaging and managing postoperative patient concerns. *Aesthetic Plast Surg* 2024; **48:**953–76.
	“AI can be used to improve healthcare access, reduce disparities, and better understand risk factors for some of the most serious diseases.”	[22] Dagli MM, Oettl FC, Gujral J, *et al.* Clinical accuracy, relevance, clarity, and emotional sensitivity of Large Language Models to surgical patient questions: Cross-Sectional study. *JMIR Form Res* 2024; **8:** e56165.
	“By simplifying complex medical terminology and presenting healthcare information in a more accessible, accurate, and relevant manner, patients can better understand their health.”	[23] Amin K, Khosla P, Doshi R, *et al.* Artificial intelligence to improve patient understanding of radiology reports. *Yale J Biol Med* 2023; **96:**407–17.
	“LLMs offer patients numerous benefits, including feeling more in control of their healthcare and understanding it better.”	[24] Blease C. Open AI meets open notes: surveillance capitalism, patient privacy and online record access. *J Med Ethics* 2024; **50:**84–9.
Potential risks and limitations of LLMs	“While LLMs often provide accurate information, cases of incomplete, unreliable, or inaccurate responses in matching with the patient’s process should not be overlooked.”	[25] Yuan J, Tang R, Jiang X, Hu X. Large Language Models for healthcare data augmentation: An example on patient-trial matching. *AMIA Annu Symp Proc* 2024; **2023:**1324–33.
	“There is a potential risk of misinformation or outdated data, as well as the loss of empathy and the inability of LLMs to fully replace consultations with healthcare professionals.”	[26] Meskó B. The impact of multimodal Large Language Models on health care’s future. *J Med Internet Res* 2023; **25:** e52865.
	“LLMs are prone to hallucinations, producing responses that seem credible but are actually incorrect.”	[27] Zhang Y, Dong Y, Mei Z, *et al.* Performance of large language models on benign prostatic hyperplasia frequently asked questions. *Prostate* 2024; **84:**807–13.
	“Racial and ethnic biases in LLM text generation for healthcare-related tasks are a major ethical concern.”	[28] Hanna JJ, Wakene AD, Lehmann CU, Medford RJ. Assessing racial and ethnic bias in text generation for healthcare-related tasks by ChatGPT1. *medRxiv* [Preprint]. 2023:2023.08.28.23294730.
	“Data privacy protection is a significant risk due to the exposure of sensitive patient data and the potential inferential disclosure of LLM results.”	[29] Wang L, Ma Y, Bi W, *et al.* An entity extraction pipeline for medical text records using Large Language Models: Analytical study. *J Med Internet Res* 2024; **26:** e54580.
	“Ethical concerns also include data integrity and the need for compliance with regulations.”	[10] Jeyaraman M, Balaji S, Jeyaraman N, Yadav S. Unraveling the ethical enigma: Artificial intelligence in healthcare. *Cureus* 2023; **15:** e43262.
Measures to be implemented to ensure reliability of health information provided by LLMs	“The path to the safe and responsible adoption of AI chatbots in healthcare should go through domain-specific training data and expert-supervised development.”	[30] Au Yeung J, Kraljevic Z, Luintel A, *et al.* AI chatbots not yet ready for clinical use. *Front Digit Health* 2023; **5:**1161098.
	“Research underscores the importance of information and proactive involvement of healthcare professionals in guiding LLM implementation.”	[31] Bhayana R, Biswas S, Cook TS, *et al.* From bench to bedside with Large Language Models: AJR expert panel narrative review. *AJR Am J Roentgenol* 2024; **223:** e2430928.
	“Further research is needed to assess patients’ attitudes toward AI-generated content, test the clarity and acceptability of responses, and determine optimal and ethical use.”	[32] Liu S, Wright AP, Mccoy AB, *et al.* Using large language model to guide patients to create efficient and comprehensive clinical care message. *J Am Med Inform Assoc* 2024; **31:**1665–70.
	“Rigorous clinical validation and testing are necessary to ensure that LLMs meet evidence-based medicine standards.”	[14] Meng X, Yan X, Zhang K, *et al.* The application of large language models in medicine: A scoping review. *iScience* 2024; **27:**109713.
	“Prompt engineering, extended query generation, and response evaluation can significantly improve the quality of results provided by LLMs.”	[17] He Z, Bhasuran B, Jin Q, *et al.* Quality of answers of generative Large Language Models vs peer users for interpreting lab test results for lay patients: Evaluation study. *J Med Internet Res* 2024; **26:** e56655.
Impact on patient/provider relationships and social implications	“LLMs offer patients an opportunity to get answers to their laboratory test interpretation questions.”	[11] Farmer H, Kreiner K, Schütz T, *et al.* The evolution of telehealth in heart failure management: The role of Large Language Models and HerzMobil as a potential use case. *Stud Health Technol Inform* 2024; **313:**228–33.
	“LLMs lack a key component of the doctor-patient encounter: empathy.”	[16] Koranteng E, Rao A, Flores E, *et al.* Empathy and equity: Key considerations for Large Language Model adoption in healthcare. *JMIR Med Educ* 2023; **9:** e51199.
	“LLM integration like ChatGPT/GPT-4 could improve medical efficiency and quality, allowing doctors more time for patient communication.”	[32] Liu S, Wright AP, Mccoy AB, *et al.* Using large language model to guide patients to create efficient and comprehensive clinical care message. *J Am Med Inform Assoc* 2024; **31:**1665–70.
	“LLMs can be used to generate follow-up questions as patients compose their messages, showing great potential to improve doctor-patient communication.”	[25] Yuan J, Tang R, Jiang X, Hu X. Large Language Models for healthcare data augmentation: An example on patient-trial matching. *AMIA Annu Symp Proc* 2024; **2023:**1324–33.
	“LLMs can alleviate administrative burdens on healthcare professionals, reduce errors, increase efficiency, and lower costs.”	[26] Meskó B. The impact of multimodal large language models on health care's future. *J Med Internet Res* 2023; **25**:e52865.
	“Relying solely on LLM recommendations without considering clinical reasoning could diminish the role of professionals in providing tailored patient care.”	[33] Peng C, Yang X, Chen A, *et al.* A study of generative large language model for medical research and healthcare. *NPJ Digit Med* 2023; **6:**210.

## Discussion

To our knowledge, this is the first review to comprehensively analyse the current state of evidence on the role and the implications of LLMs like ChatGPT in public health, offering a perspective which takes in account both risks and benefits for patient and population use.

The discussion follows the four main predefined thematic categories which guided the conduct of the review.

It appears that the adoption of LLMs in healthcare is reshaping how patients access medical information, offering a new pathway for democratizing health knowledge by simplifying complex concepts and providing quick, user-friendly explanations. This can be particularly advantageous for populations with limited access to traditional healthcare services or those who struggle to understand medical jargon. LLMs could contribute to more informed decision-making and foster patient empowerment, helping to change the role of patients from passive recipients to active participants and reshaping the traditional patient-healthcare professional hierarchy and relationship. Healthcare professionals may perceive this scenario as an opportunity to facilitate a more dynamic and interactive communication and support their role [32], but may also be concerned about patients’ overreliance on AI-provided information without appropriate medical oversight.

Consistently, the review highlights significant concerns associated with the use of LLMs in healthcare which should not be underestimated. These include issues of misinformation or biased content. Notably, LLMs can produce very convincing and apparently well-argued but incorrect or misleading answers. This otherwise unpredictable behavior is critical when patients may rely heavily on AI responses. Instances of hallucinations pose a threat to patient safety [15, 25]. Additionally, concerns about algorithmic bias can exacerbate health disparities, especially when the models are trained on data that may not represent the diversity of the patient population [16, 28]. Furthermore, data privacy concerns remain a significant barrier, as the use of patient information in AI training processes could lead to potential breaches of confidentiality [9, 34]. As synthetic data generated by AI models are trained on real-world datasets, they may suffer from and perpetuate underlying biases, incompleteness or faults. This may lead to incorrect or misleading outcomes, potentially reinforcing systemic biases that affect specific patient groups.

These findings suggest the need to implement appropriate measures, e.g. stringent regulatory frameworks, to ensure the responsible and ethical use of AI in healthcare settings. In this regard, it is worth mentioning the World Health Organization’s document *Ethics and governance of artificial intelligence for health. Guidance on large multi-modal models*, which provides a comprehensive framework to guide the ethical deployment of LLMs in healthcare, ensuring that they benefit all individuals equitably while minimizing potential risks [35].

The findings of this review underscore the importance of using high-quality and healthcare professional-controlled training data and involving clinical experts throughout the development and implementation phases of LLMs to ensure the reliability and accuracy of their outputs [14, 30, 36]. Additionally, integrating real-time monitoring systems and transparent reporting mechanisms could help mitigate the risks of misinformation and biases [16, 36].

Considering the impact of LLMs into patient care, it has the potential to enhance efficiency and streamline certain aspects of medical consultations, yet it also poses risks to the human element of healthcare. The impersonality of AI-generated responses may detract from the empathetic support that is central to the patient-provider relationship. While LLMs can assist in answering routine questions and providing follow-up information, they cannot replace the nuanced understanding and emotional intelligence of a human clinician [16].

“Artificial empathetic language” can be recognized from real empathy and should be complementary, not a substitute for human empathy, to avoid further isolating patients and compromising the therapeutic alliance [16]. As AI tools become more prevalent, it is crucial to find a balance that leverages their strengths without eroding the fundamental trust and communication that characterize effective healthcare.

A potential drawback of the growing use of LLMs in healthcare is the risk of deskilling among healthcare professionals, who may gradually lose essential clinical and cognitive skills as they increasingly rely on AI systems for complex decision-making tasks [37]. It is essential to remain consistently aware of these limitations, ensuring that AI integration enhances the human element in healthcare.

An important consideration when analysing the use of ChatGPT and other LLMs in healthcare is that, as these models are not specifically trained in the medical field, they may sometimes provide responses that are not fully accurate or suitable for healthcare purposes. This raises questions about their reliability in clinical settings, where the accuracy and precision of information are crucial.

However, specialized LLMs tailored for the healthcare sector are being developed, focusing on medical knowledge and clinical guidelines. These models, if trained on large volumes of specific, relevant data, can deliver more accurate and contextually appropriate responses, enhancing the quality of information available to patients and assisting healthcare professionals in diagnosis, treatment planning, and patient communication [38, 39].

This study has several strengths. While other previous reviews were limited to specific fields of medicine, often concentrating on specialized aspects [31] or narrowly defined applications of AI within medical contexts [11], in our investigation we intended to adopt a broader perspective. In formulating the PECO question we aimed to implement an inclusive approach, encompassing both direct users of AI-driven LLMs, such as patients and healthcare professionals, and the general population, and to achieve a deeper understanding of the phenomenon’s complexity and the evaluation of individual and collective impacts. Furthermore, it considers among the outcomes which measures could improve or ensure accuracy of health information, a critical factor in a context where inaccuracies or misinformation can significantly impact public health.

One significant limitation of this review is its narrative design, which was chosen to match the heterogeneous nature of the available data. The field of LLMs is rapidly evolving, with frequent changes in models, applications, and training data. Consequently, the selection of sources may have been influenced by temporal biases due to the rapid development of the field, potentially excluding more recent and relevant advancements. Additionally, the data used to train LLMs may contain intrinsic biases, such as cultural, demographic, or geographical distortions, which could be reflected in the responses generated by the models, limiting the reliability and generalizability of the information.

Another aspect that could have introduced bias is the selection of studies. The search was primarily conducted in PubMed, which, while a reliable source, may not have included all relevant publications, particularly those from other data sources or non-indexed journals. Moreover, the diversity of the LLMs’ user base, which includes citizens and patients with varying experiences and information needs, could have influenced the way AI was interacted with, creating cognitive biases in the data analysis. For instance, user approaches may vary based on sociodemographic factors, influencing the quality of the responses generated by the models.

Furthermore, our analysis primarily focused on studies from countries like the United States (65 studies), Europe (19 studies), and Asia (15 studies), which may not fully represent the global diversity in the perception and use of LLMs. This could have introduced a geographical bias, limiting the generalizability of the findings to other healthcare contexts.

Finally, the rapid evolution of LLM technology and applications may mean that the findings of this review will become outdated in a relatively short period, limiting the longevity of the conclusions drawn. While this review reflects the current state of the art, the findings could significantly change if reassessed in the future as the models evolve and are applied in different contexts. Despite these limitations, we believe that documenting the current state of LLM use in healthcare is essential, and this review represents an important step in guiding future developments and applications.

In conclusion, the results of this narrative review suggest that the use of LLMs in healthcare represents a significant shift in how patients access and interpret health information. While their potential for improved access is substantial, it is accompanied by critical risks and limitations that must be approached with caution. Implementing robust safeguards and involving clinicians in the oversight of these technologies will be essential to harness their benefits while mitigating the risks and ensure patient safety, ethical standards, and the preservation of the core values of the doctor-patient relationship. A balanced, patient-centered approach that integrates LLMs as a complementary tool, rather than a replacement for human expertise, is essential for their incorporation into the healthcare ecosystem.

## Supplementary Material

ckaf189_Supplementary_Data

## Data Availability

The datasets used and/or analysed during the current study are available in the Supplementary Material of this article or from the corresponding author on reasonable request. Key points This is the first narrative review on citizens’ and patients’ perceptions about the use of large language models (LLMs) in health information. LLMs provide improved speed and sufficient accuracy in diagnosis and can reduce inequalities in health information and simplify complexity. LLMs carry the potential risk of incomplete, unreliable, inaccurate, incorrect, and/or outdated health information and the possibility of an inadequate privacy protection. A safe and responsible adoption of AI chatbots in healthcare implies a careful and intensive training under the supervision of experienced clinicians. The use of LLMs entails a loss of the empathy typical of the interhuman physician-patient relationship.
