# Supplementary material for: The use of artificial intelligence in healthcare as perceived by the citizens and patients: a narrative review of the literature
Source: Eur J Public Health. 2025 Nov 14;35(6):1092–9. doi: 10.1093/eurpub/ckaf189 (PMC12707470; doi:10.1093/eurpub/ckaf189)
Supplement: ckaf189_Supplementary_Data [file ckaf189_supplementary_data.docx]

| **Table S1. List of the 120 studies included into the analysis** | |
| --- | --- |
| 1 | Abbasian M, Khatibi E, Azimi I, et al. Foundation metrics for evaluating effectiveness of healthcare conversations powered by generative AI. *NPJ Digit Med* 2024;**7:**82. |
| 2 | Abi-Rafeh J, Mroueh VJ, Bassiri-Tehrani B, et al. Complications following body contouring: Performance validation of bard, a novel AI Large Language Model, in triaging and managing postoperative patient concerns. *Aesthetic Plast Surg* 2024;**48:**953–76. |
| 3 | Amin K, Khosla P, Doshi R, et al. Artificial intelligence to improve patient understanding of radiology reports. *Yale J Biol Med* 2023;**96:**407–17. |
| 4 | Andrew A. Potential applications and implications of large language models in primary care. *Fam Med Community Health* 2024;**12:**e002602. |
| 5 | Au Yeung J, Kraljevic Z, Luintel A, et al. AI chatbots not yet ready for clinical use. *Front Digit Health* 2023;**5:**1161098. |
| 6 | Balas M, Wadden JJ, Hébert PC, et al. Exploring the potential utility of AI large language models for medical ethics: an expert panel evaluation of GPT-4. *J Med Ethics* 2024;**50:**90–6. |
| 7 | Baxter SL, Longhurst CA, Millen M, et al. Generative artificial intelligence responses to patient messages in the electronic health record: early lessons learned. *JAMIA Open* 2024;**7:**ooae028. |
| 8 | Benary M, Wang XD, Schmidt M, et al. Leveraging Large Language Models for decision support in personalized oncology. *JAMA Netw Open* 2023;**6:**e2343689. |
| 9 | Bernstein IA, Zhang YV, Govil D, et al. Comparison of ophthalmologist and Large Language Model chatbot responses to online patient eye care questions. *JAMA Netw Open* 2023;**6:**e2330320. |
| 10 | Bhayana R, Biswas S, Cook TS, et al. From bench to bedside with Large Language Models: AJR expert panel narrative review. *AJR Am J Roentgenol* 2024;**223:**e2430928. |
| 11 | Blease C. Open AI meets open notes: Surveillance capitalism, patient privacy and online record access. *J Med Ethics* 2024;**50:**84–9. |
| 12 | Bricker JB, Sullivan B, Mull K, et al. Conversational Chatbot for cigarette smoking cessation: Results from the 11-step user-centered design development process and randomized controlled trial. *JMIR Mhealth Uhealth* 2024;**12:**e57318. |
| 13 | Chari S, Acharya P, Gruen DM, et al. Informing clinical assessment by contextualizing post-hoc explanations of risk prediction models in type-2 diabetes. *Artif Intell Med* 2023;**137:**102498. |
| 14 | Chen Z, Wang Q, Sun Y, et al. Chat-ePRO: Development and pilot study of an electronic patient-reported outcomes system based on ChatGPT. *J Biomed Inform* 2024;154:104651. |
| 15 | Choudhury A, Chaudhry Z. Large Language Models and user trust: Consequence of self-referential learning loop and the deskilling of health care professionals. *J Med Internet Res* 2024;**26:**e56764. |
| 16 | Cohen IG. What should ChatGPT mean for bioethics? *Am J Bioeth* 2023;**23:**8–16. |
| 17 | Cohen SA, Brant A, Fisher AC, et al. Dr. Google vs. Dr. ChatGPT: Exploring the use of artificial intelligence in ophthalmology by comparing the accuracy, safety, and readability of responses to frequently asked patient questions regarding cataracts and cataract surgery. *Semin Ophthalmol* 2024;**39:**472–79. |
| 18 | Cung M, Sosa B, Yang HS, et al. The performance of artificial intelligence chatbot large language models to address skeletal biology and bone health queries. *J Bone Miner Res* 2024 Mar 22;**39:**106–15. |
| 19 | Dagli MM, Oettl FC, Gujral J, et al. Clinical accuracy, relevance, clarity, and emotional sensitivity of Large Language Models to surgical patient questions: Cross-Sectional study. *JMIR Form Res* 2024;**8:**e56165. |
| 20 | Delsoz M, Madadi Y, Raja H, et al. Performance of ChatGPT in diagnosis of corneal eye diseases. *Cornea* 2024;**43:**664–70. |
| 21 | Denecke K, May R; LLMHealthGroup; Rivera Romero O. Potential of Large Language Models in health care: Delphi study. *J Med Internet Res* 2024;**26:**e52399. |
| 22 | Eggmann F, Weiger R, Zitzmann NU, Blatz MB. Implications of large language models such as ChatGPT for dental medicine. *J Esthet Restor Dent* 2023;**35:**1098–102. |
| 23 | Eggmann F, Blatz MB. ChatGPT: Chances and challenges for dentistry. *Compend Contin Educ Den* 2023;**44:**220–4. |
| 24 | Farmer H, Kreiner K, Schütz T, et al. The evolution of telehealth in heart failure management: The role of Large Language Models and HerzMobil as a potential use case. *Stud Health Technol Inform* 2024;**313:**228–33. |
| 25 | Ferrario A, Sedlakova J, Trachsel M. The role of humanization and robustness of Large Language Models in conversational artificial intelligence for individuals with depression: A critical analysis. *JMIR Ment Health* 2024;**11:**e56569. |
| 26 | Fraser H, Crossland D, Bacher I, Ranney M, Madsen T, Hilliard R. Comparison of Diagnostic and Triage Accuracy of Ada Health and WebMD Symptom Checkers, ChatGPT, and Physicians for Patients in an Emergency Department: Clinical Data Analysis Study. JMIR Mhealth Uhealth. 2023;**11:**e49995. |
| 27 | Ge J, Sun S, Owens J,et al. Development of a liver disease-specific large language model chat interface using retrieval-augmented generation. *Hepatology* 2024;**80:**1158–68. |
| 28 | Giannakopoulos K, Kavadella A, Aaqel Salim A, et al. Evaluation of the performance of generative AI Large Language Models ChatGPT, Google Bard, and Microsoft Bing Chat in supporting evidence-based dentistry: Comparative mixed methods study. *J Med Internet Res* 2023;**25:**e51580. |
| 29 | Giuffrè M, Kresevic S, You K, et al. Systematic review: The use of large language models as medical chatbots in digestive diseases. *Aliment Pharmacol Ther* 2024;**60:**144–66. |
| 30 | Giuffrè M, Kresevic S, Pugliese N, et al. Optimizing large language models in digestive disease: strategies and challenges to improve clinical outcomes. *Liver Int* 2024;**44:**2114–24. |
| 31 | Goh E, Bunning B, Khoong E, et al. ChatGPT influence on medical decision-making, bias, and equity: A randomized study of clinicians evaluating clinical vignettes. *medRxiv* [Preprint] 2023;2023.11.24.23298844. |
| 32 | Goh E, Gallo R, Hom J, et al. Influence of a Large Language Model on diagnostic reasoning: A randomized clinical vignette study. *JAMA Netw Open* 2024;**7:**e2440969. |
| 33 | González-Palau F, Franco M, Bamidis P, et al. The effects of a computer-based cognitive and physical training program in a healthy and mildly cognitive impaired aging sample. *Aging Ment Health* 2014;**18:**838–46. |
| 34 | Guillen-Grima F, Guillen-Aguinaga S, Guillen-Aguinaga L, et al. Evaluating the efficacy of ChatGPT in navigating the Spanish Medical Residency Entrance Examination (MIR): Promising horizons for AI in clinical medicine. *Clin Pract* 2023;**13:**1460–87. |
| 35 | Hadar-Shoval D, Asraf K, Mizrachi Y, et al. Assessing the alignment of Large Language Models with human values for mental health integration: Cross-sectional study using Schwartz's theory of basic values. *JMIR Ment Health* 2024;**11:**e55988. |
| 36 | Hanna JJ, Wakene AD, Lehmann CU, Medford RJ. Assessing racial and ethnic bias in text generation for healthcare-related tasks by ChatGPT1. *medRxiv* [Preprint]. 2023:2023.08.28.23294730. |
| 37 | Healey E, Tan A, Flint K, et al. Leveraging Large Language Models to analyze continuous glucose monitoring data: A case study. *medRxiv* [Preprint] 2024:2024.04.06.24305022. |
| 38 | Healey E, Tan A, Flint K, Ruiz J, Kohane I. Leveraging Large Language Models to Analyze Continuous Glucose Monitoring Data: A Case Study. medRxiv [Preprint]. 2024 Apr 8:2024.04.06.24305022. |
| 39 | Hirosawa T, Harada Y, Tokumasu K, et al. Evaluating ChatGPT-4's diagnostic accuracy: Impact of visual data integration. *JMIR Med Inform* 2024;**12:**e55627. |
| 40 | Hosseini M, Gao CA, Liebovitz DM, et al. An exploratory survey about using ChatGPT in education, healthcare, and research. *PLoS One* 2023;**18:**e0292216. |
| 41 | Hueso M, Álvarez R, Marí D, eet al. Is generative artificial intelligence the next step toward a personalized hemodialysis? *Rev Invest Clin* 2023;**75:**309–17. |
| 42 | Huo B, Calabrese E, Sylla P, et al. The performance of artificial intelligence large language model-linked chatbots in surgical decision-making for gastroesophageal reflux disease. *Surg Endosc* 2024;**38:**2320–30. |
| 43 | Jeyaraman M, Balaji S, Jeyaraman N, Yadav S. Unraveling the ethical enigma: Artificial intelligence in healthcare. *Cureus* 2023;**15:**e43262. |
| 44 | Jin Q, Wang Z, Floudas CS, et al. Matching patients to clinical trials with large language models. *Nat Commun* 2024;**15:**9074. |
| 45 | Kim S, Kim K, Wonjeong Jo C. Accuracy of a large language model in distinguishing anti- and pro-vaccination messages on social media: The case of human papillomavirus vaccination. *Prev Med Rep* 2024;**42:**102723. |
| 46 | Koranteng E, Rao A, Flores E, et al. Empathy and equity: Key considerations for Large Language Model adoption in health care. *JMIR Med Educ* 2023;**9:**e51199. |
| 47 | Kozaily E, Geagea M, Akdogan ER, et al. Accuracy and consistency of online large language model-based artificial intelligence chat platforms in answering patients' questions about heart failure. *Int J Cardiol* 2024;**408:**132115. |
| 48 | Krishnamoorthy R, Nagarajan V, Pour H, et al. Voice-Enabled Response Analysis Agent (VERAA): Leveraging Large Language Models to map voice responses in SDoH survey. *AMIA Jt Summits Transl Sci Proc* 2024;**2024:**258–65. |
| 49 | Kuckelman IJ, Yi PH, Bui M, et al. Assessing AI-powered patient education: A case study in radiology. *Acad Radiol* 2024;**31:**338–42. |
| 50 | Kumar RP, Sivan V, Bachir H, et al. Can artificial intelligence mitigate missed diagnoses by generating differential diagnoses for neurosurgeons? *World Neurosurg* 2024;**187:**e1083–8. |
| 51 | Laios A, Theophilou G, De Jong D, Kalampokis E. The Future of AI in Ovarian Cancer Research: The Large Language Models Perspective. Cancer Control. 2023;**30:**10732748231197915. |
| 52 | Lambert R, Choo ZY, Gradwohl K, et al. Assessing the application of Large Language Models in generating dermatologic patient education materials according to reading level: Qualitative study. *JMIR Dermatol* 2024;**7:**e55898. |
| 53 | Law S, Oldfield B, Yang W; Global Obesity Collaborative. ChatGPT/GPT-4 (large language models): Opportunities and challenges of perspective in bariatric healthcare professionals. *Obes Rev* 2024;**25:**e13746. |
| 54 | Lee JH, Choi E, McDougal R, Lytton WW. GPT-4 performance for neurologic localization. *Neurol Clin Pract* 2024;**14:**e200293. |
| 55 | Lim B, Seth I, Cuomo R, et al. Can AI answer my questions? Utilizing artificial intelligence in the perioperative assessment for abdominoplasty patients. *Aesthetic Plast Surg* 2024;**48:**4712–24. |
| 56 | Lim ZW, Pushpanathan K, Yew SME, et al. Benchmarking large language models' performances for myopia care: a comparative analysis of ChatGPT-3.5, ChatGPT-4.0, and Google Bard. *EBioMedicine* 2023;**95:**104770. |
| 57 | Liu S, Wright AP, Mccoy AB, et al. Using large language model to guide patients to create efficient and comprehensive clinical care message. *J Am Med Inform Assoc* 2024;**31:**1665–70. |
| 58 | Longwell JB, Hirsch I, Binder F, et al. Performance of Large Language Models on medical oncology examination questions. *JAMA Netw Open* 2024;**7:**e2417641. |
| 59 | López-Úbeda P, Martín-Noguerol T, Díaz-Angulo C, Luna A. Evaluation of large language models performance against humans for summarizing MRI knee radiology reports: A feasibility study. *Int J Med Inform* 2024;**187:**105443. |
| 60 | Madadi Y, Delsoz M, Lao PA, et al. ChatGPT assisting diagnosis of neuro-ophthalmology diseases based on case reports. *medRxiv* [Preprint] 2023:2023.09.13.23295508. |
| 61 | Makrygiannakis MA, Giannakopoulos K, Kaklamanos EG. Evidence-based potential of generative artificial intelligence large language models in orthodontics: a comparative study of ChatGPT, Google Bard, and Microsoft Bing. *Eur J Orthod* 2024:cjae017. |
| 62 | Mannstadt I, Goodman SM, Rajan M, et al. A novel approach for mixed-methods research using Large Language Models: A report using patients' perspectives on barriers to arthroplasty. *ACR Open Rheumatol* 2024;**6:**375–9. |
| 63 | Masanneck L, Schmidt L, Seifert A, et al. Triage performance across Large Language Models, ChatGPT, and untrained doctors in emergency medicine: Comparative study. *J Med Internet Res* 2024;**26:**e53297. |
| 64 | Mashatian S, Armstrong DG, Ritter A, et al. Building trustworthy generative srtificial intelligence for diabetes care and limb preservation: A medical knowledge extraction case. J Diabetes Sci Technol 2024;**19:**1264–70. |
| 65 | Meng X, Yan X, Zhang K, et al. The application of large language models in medicine: A scoping review. *IScience* 2024;**27:**109713. |
| 66 | Menz BD, Kuderer NM, Bacchi S, et al. Current safeguards, risk mitigation, and transparency measures of large language models against the generation of health disinformation: repeated cross sectional analysis. *BMJ* 2024;**384:**e078538. |
| 67 | Mesko B. The ChatGPT (Generative Artificial Intelligence) revolution has made artificial intelligence approachable for medical professionals. *J Med Internet Res* 2023;**25:**e48392. |
| 68 | Meskó B. The impact of multimodal Large Language Models on health care's future. *J Med Internet Res* 2023;**25:**e52865. |
| 69 | Mira FA, Favier V, Dos Santos Sobreira Nunes H, et al. Chat GPT for the management of obstructive sleep apnea: do we have a polar star?. *Eur Arch Otorhinolaryngol* 2024;**281:**2087–93. |
| 70 | Mohamed AA, Lucke-Wold B. Text-to-video generative artificial intelligence: sora in neurosurgery. *Neurosurg Rev* 2024;**47:**272. |
| 71 | Mondal H, Dash I, Mondal S, Behera JK. ChatGPT in answering queries related to lifestyle-related diseases and disorders. *Cureus* 2023;**15:**e48296. |
| 72 | Moura L, Jones DT, Sheikh IS, et al. Implications of Large Language Models for quality and efficiency of neurologic care: Emerging issues in neurology. *Neurology* 2024;**102:**e209497. |
| 73 | Naqvi WM, Shaikh SZ, Mishra GV. Large language models in physical therapy: time to adapt and adept. *Front Public Health* 2024;**12:**1364660. |
| 74 | Naz R, Akacı O, Erdoğan H, Açıkgöz A. Can large language models provide accurate and quality information to parents regarding chronic kidney diseases?. *J Eval Clin Pract* 2024;**30:**1556–64. |
| 75 | Neo JRE, Ser JS, Tay SS. Use of large language model-based chatbots in managing the rehabilitation concerns and education needs of outpatient stroke survivors and caregivers. *Front Digit Health* 2024;**6:**1395501. |
| 76 | Nielsen JPS, von Buchwald C, Grønhøj C. Validity of the large language model ChatGPT (GPT4) as a patient information source in otolaryngology by a variety of doctors in a tertiary otorhinolaryngology department. *Acta Otolaryngol* 2023;**143:**779–82. |
| 77 | Nievas M, Basu A, Wang Y, Singh H. Distilling large language models for matching patients to clinical trials. *J Am Med Inform Assoc* 2024;**31:**1953–63. |
| 78 | Ong H, Ong J, Cheng R, et al. GPT Technology to help address longstanding barriers to care in free medical clinics. Ann Biomed Eng 2023;**51:**1906–9. |
| 79 | Park YJ, Pillai A, Deng J, et al. Assessing the research landscape and clinical utility of large language models: a scoping review. *BMC Med Inform Decis Mak* 2024;24:72. |
| 80 | Parsa S, Somani S, Dudum R, Jain SS, Rodriguez F. Artificial Intelligence in Cardiovascular Disease Prevention: Is it Ready for Prime Time?. *Current Atherosclerosis Reports* 2024;**26:**263–72. |
| 81 | Peng C, Yang X, Chen A, et al. A study of generative large language model for medical research and healthcare. *NPJ Digit Med* 2023;**6:**210. |
| 82 | Preiksaitis C, Ashenburg N, Bunney G, et al. The role of Large Language Models in transforming emergency medicine: Scoping review. *JMIR Med Inform* 2024;**12:**e53787. |
| 83 | Pugliese G, Maccari A, Felisati E, et al. Are artificial intelligence large language models a reliable tool for difficult differential diagnosis? An a posteriori analysis of a peculiar case of necrotizing otitis externa. *Clin Case Rep* 2023;**11:**e7933. |
| 84 | Rahimli Ocakoglu S, Coskun B. The emerging role of AI in patient education: A comparative analysis of LLM accuracy for pelvic organ prolapse. *Med Princ Pract* 2024;**33:**330–7. |
| 85 | Romanopoulou ED, Zilidou VI, Gilou S, et al. Technology enhanced health and social care for vulnerable people during the COVID-19 outbreak. *Front Hum Neurosci* 2021;**15:**721065. |
| 86 | Sallam M. ChatGPT utility in healthcare education, research, and practice: Systematic review on the promising perspectives and valid concerns. *Healthcare (Basel)* 2023;**11:**887. |
| 87 | Samsonov AP, Habibi A, Butler JJ, et al. Artificial Intelligence Language Models are useful tools for patients undergoing total ankle replacement. *Foot Ankle Spec* 2024:19386400241249810. |
| 88 | Savage T, Wang J, Shieh L. A Large Language Model screening tool to target patients for best practice alerts: Development and validation. *JMIR Med Inform* 2023;**11:**e49886. |
| 89 | Savage T, Nayak A, Gallo R, et al. Diagnostic reasoning prompts reveal the potential for large language model interpretability in medicine. *NPJ Digit Med* 2024;**7:**20. |
| 90 | Sezgin E. Redefining virtual assistants in health care: The future with Large Language Models. *J Med Internet Res* 2024;**26:**e53225. |
| 91 | Shahab O, El Kurdi B, Shaukat A, eet al. Large language models: a primer and gastroenterology applications. *Therap Adv Gastroenterol* 2024;**17:**17562848241227031. |
| 92 | Skryd A, Lawrence K. ChatGPT as a tool for medical education and clinical decision-making on the ards: Case study. *JMIR Form Res* 2024;**8:**e51346. |
| 93 | Sorrentino C, Canoro V, Russo M, et al. Assessing ChatGPT ability to answer frequently asked questions about essential tremor. *Tremor Other Hyperkinet Mov* (NY) 2024;**14:**33. |
| 94 | Sridharan K, Sivaramakrishnan G. Enhancing readability of USFDA patient communications through large language models: a proof-of-concept study. *Expert Rev Clin Pharmacol* 2024;**17:**731–41. |
| 95 | Srinivasan N, Samaan JS, Rajeev ND, et al. Large language models and bariatric surgery patient education: A comparative readability analysis of GPT-3.5, GPT-4, Bard, and online institutional resources. *Surg Endosc* 2024;**38:**2522–32. |
| 96 | Stroop A, Stroop T, Zawy Alsofy S, et al. Large language models: Are artificial intelligence-based chatbots a reliable source of patient information for spinal surgery?. *Eur Spine J* 2024;**33:**4135–43. |
| 97 | Tailor PD, Xu TT, Fortes BH, et al. Appropriateness of ophthalmology recommendations from an online chat-based artificial intelligence model. *Mayo Clin Proc Digit Health* 2024;**2:**119–8. |
| 98 | Tan TF, Thirunavukarasu AJ, Campbell JP, et al. Generative artificial intelligence through ChatGPT and other Large Language Models in ophthalmology: Clinical applications and challenges. *Ophthalmol Sci* 2023;**3:**100394. |
| 99 | Temsah MH, Jamal A, Alhasan K, et al. Transforming virtual healthcare: The potentials of ChatGPT-4omni in telemedicine. *Cureus* 2024;**16:**e61377. |
| 100 | Tharakan S, Klein B, Bartlett L, et al Do ChatGPT and Google differ in answers to commonly asked patient questions regarding total shoulder and total elbow arthroplasty?. *J Shoulder Elbow Surg* 2024;**33:**e429–7. |
| 101 | Thirunavukarasu AJ. How can the clinical aptitude of AI assistants be assayed?. *J Med Internet Res* 2023;**25:**e51603. |
| 102 | Truhn D, Weber CD, Braun BJ, et al. A pilot study on the efficacy of GPT-4 in providing orthopedic treatment recommendations from MRI reports. *Sci Rep* 2023;**13:**20159. |
| 103 | Ullah E, Parwani A, Baig MM, Singh R. Challenges and barriers of using large language models (LLM) such as ChatGPT for diagnostic medicine with a focus on digital pathology - a recent scoping review. *Diagn Pathol* 2024;**19:**43. |
| 104 | Vaid A, Duong SQ, Lampert J, et al. Local large language models for privacy-preserving accelerated review of historic echocardiogram reports. *J Am Med Inform Assoc* 2024;**31:**2097–102. |
| 105 | van Diessen E, van Amerongen RA, Zijlmans M, Otte WM. Potential merits and flaws of large language models in epilepsy care: A critical review. *Epilepsia* 2024;**65:**873–86. |
| 106 | Venerito V, Iannone F. Large language model-driven sentiment analysis for facilitating fibromyalgia diagnosis. *RMD Open* 2024;**10:**e004367. |
| 107 | Wang A, Wu Y, Ji X, et al. Assessing and optimizing Large Language Models on spondyloarthritis multi-choice question answering: Protocol for enhancement and assessment. *JMIR Res Protoc* 2024;**13:**e57001. |
| 108 | Wang H, Gao C, Dantona C, et al. DRG-LLaMA : tuning LLaMA model to predict diagnosis-related group for hospitalized patients. *NPJ Digit Med* 2024;7:16. |
| 109 | Wang L, Ma Y, Bi W, et al. An entity extraction pipeline for medical text records using Large Language Models: Analytical study. *J Med Internet Res* 2024;**26:**e54580. |
| 110 | Wang L, Wan Z, Ni C, et al. A systematic review of ChatGPT and other conversational Large Language Models in healthcare. *J Med Internet Res* 2024;**26:**e22769. |
| 111 | Wei Q, Yao Z, Cui Y, et al. Evaluation of ChatGPT-generated medical responses: A systematic review and meta-analysis. *J Biomed Inform* 2024;**151:**104620. |
| 112 | Williams CYK, Zack T, Miao BY, et al. Use of a Large Language Model to assess clinical acuity of adults in the emergency department. *JAMA Netw Open* 2024;**7:**e248895. |
| 113 | Wu G, Zhao W, Wong A, Lee DA. Patients with floaters: Answers from virtual assistants and large language models. *Digit Health* 2024;**10:**20552076241229933. |
| 114 | Xu X, Chen Y, Miao J. Opportunities, challenges, and future directions of large language models, including ChatGPT in medical education: a systematic scoping review. *J Educ Eval Health Prof* 2024;**21:**6. |
| 115 | Yalamanchili A, Sengupta B, Song J, et al. Quality of Large Language Model responses to radiation oncology patient care questions. *JAMA Netw Open* 2024;**7:**e244630. |
| 116 | Yuan J, Tang R, Jiang X, Hu X. Large Language Models for healthcare data augmentation: An example on patient-trial matching. *AMIA Annu Symp Proc* 2024;**2023:**1324–33. |
| 117 | Zaretsky J, Kim JM, Baskharoun S, et al. Generative artificial intelligence to transform inpatient discharge summaries to patient-friendly language and format. *JAMA Netw Open* 2024;**7:**e240357. |
| 118 | Zernikow J, Grassow L, Gröschel J, et al. [Clinical application of large language models : Does ChatGPT replace medical report formulation? An experience report]. *Inn Med (Heidelb)* 2023;**64:**1058–64. |
| 119 | Zhang Y, Dong Y, Mei Z, et al. Performance of large language models on benign prostatic hyperplasia frequently asked questions. *Prostate* 2024;**84:**807–13. |
| 120 | Zhou S, Luo X, Chen C, et al. The performance of large language model-powered chatbots compared to oncology physicians on colorectal cancer queries. *Int J Surg* 2024;**110:**6509–17. |
